# A Case of Cecal Volvulus Successfully Managed With Transnasal Ileus Tube Decompression Followed by Elective Laparoscopic Ileocecal Resection

**DOI:** 10.7759/cureus.91328

**Published:** 2025-08-31

**Authors:** Masayoshi Hirohara, Hiroyoshi Tsuchida, Shuichiro Uemura, Yuhi Ozaki, Shin Saida, Nobusada Koike

**Affiliations:** 1 Gastrointestinal Surgery, Hachioji Digestive Disease Hospital, Hachioji, JPN

**Keywords:** cecal volvulus, colon volvulus repair surgery, decompression tube, ileocecal resection, laparoscopic repair

## Abstract

Cecal volvulus is a rare but potentially life-threatening cause of colonic obstruction. Emergency surgery is standard but is associated with high morbidity and mortality, especially in high-risk patients. Therefore, non-operative management may be an important alternative in selected cases.

A 90-year-old man presented to the emergency department with abdominal distension and pain. He was diagnosed with cecal volvulus without evidence of intestinal necrosis, accompanied by right lower lobe atelectasis and pleural effusion, both attributed to compression of the lung secondary to massive colonic distension. Given the absence of necrosis and the high anesthetic risk associated with advanced age and underlying pulmonary compromise, non-operative management was initiated using a transnasal ileus tube. Successful detorsion was confirmed on day 7 post-insertion, and the patient was discharged. Elective laparoscopic ileocecal resection was performed subsequently to prevent recurrence. Postoperative pneumonia was noted, but no other complications occurred, and the patient was discharged on postoperative day 19. At the 11-month follow-up, the patient remained in good health with no evidence of recurrence.

We report a case of cecal volvulus without intestinal necrosis successfully managed by transnasal ileus tube decompression followed by elective laparoscopic ileocecal resection. This approach may be a viable option to avoid emergency surgery in high-risk patients and expands the therapeutic options for elderly individuals.

## Introduction

Cecal volvulus is the second most common type of colonic volvulus, following sigmoid volvulus, and typically occurs due to inadequate fixation of the cecum to the posterior abdominal wall [1]. Emergency surgery is generally required for cecal volvulus to prevent further ischemia and recurrence. On the other hand, non-operative approaches such as colonoscopic reduction or decompression via a transanal ileus tube have been successful in cases without signs of intestinal necrosis [2,3]. However, reports of successful non-operative management of cecal volvulus using a transnasal ileus tube remain limited, highlighting the rarity of this approach. Emergency surgeries are associated with higher morbidity and mortality rates, particularly in patients considered high risk for undergoing general anesthesia. Thus, whenever feasible, elective surgery is preferred to mitigate these risks.

Here, we report a case illustrating the successful application of this strategy in a high-risk elderly patient, demonstrating its potential as an alternative to emergency surgery. We present a case of a 90-year-old male patient with cecal volvulus who did not exhibit signs of intestinal ischemia. He developed right lower lobe atelectasis and pleural effusion caused by compression of the lung due to massive colonic distension. The condition was successfully managed with decompression using a transnasal ileus tube, followed by elective laparoscopic right hemicolectomy to prevent recurrence.

## Case presentation

**Figure 1:**
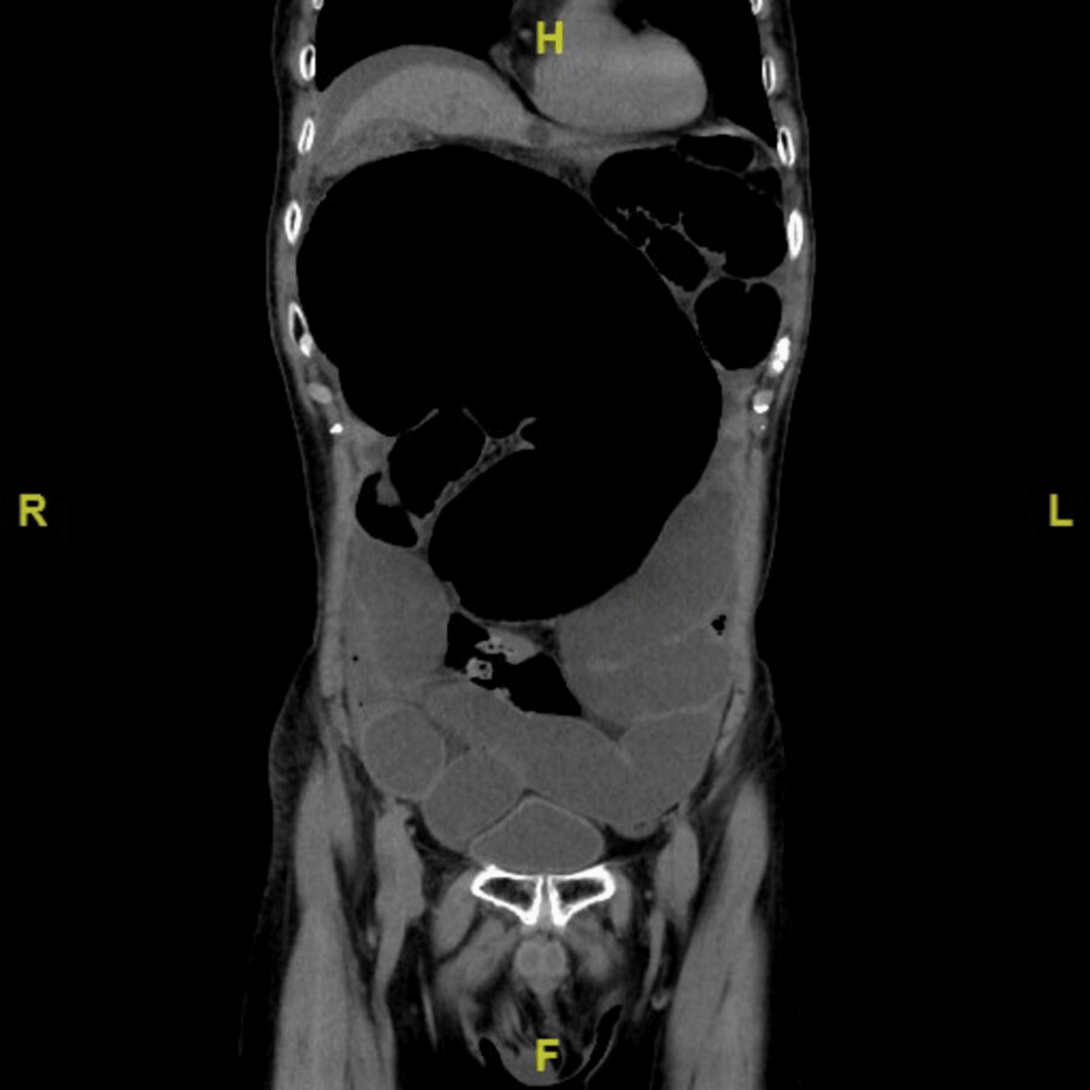
CT scan showed cecal dilation consistent with cecal volvulus Non-contrast CT on admission showing marked cecal dilatation consistent with cecal volvulus. No free air or signs of bowel necrosis are observed.

**Figure 2:**
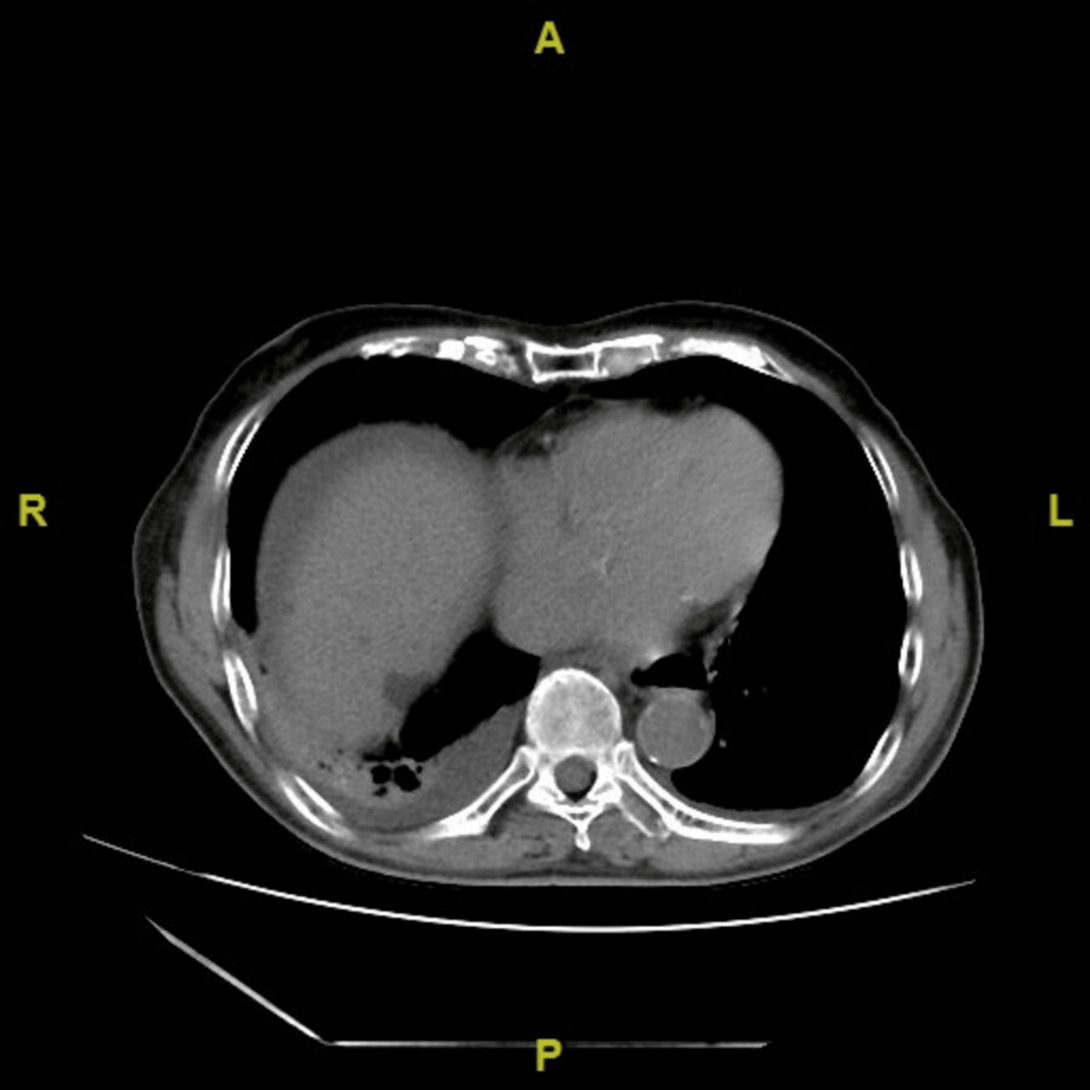
CT scan showed right lower lobe atelectasis and pleural effusion Non-contrast CT on admission showing right lower lobe atelectasis and pleural effusion. These findings contributed to the patient’s increased perioperative risk.

A 90-year-old man presented to the emergency department in September 2024 with a two-day history of abdominal distension and pain. His medical history included hypertension, dyslipidemia, chronic constipation, and a laparoscopic cholecystectomy two years prior. Upon arrival at the emergency department, vital signs were stable (BP 119/69 mmHg, HR 90 bpm, temp 36.8°C, RR 18/min). Physical examination revealed generalized abdominal distension with mild epigastric tenderness without rebound tenderness. Blood tests revealed a mildly elevated CRP level of 34.1 mg/L, while white blood cell count (7.13 ×10⁹/L) and lactate dehydrogenase (2.65 μkat/L) were within normal ranges, supporting the absence of bowel necrosis (Table 1). A CT scan showed cecal dilation consistent with cecal volvulus, accompanied by right lower lobe atelectasis and pleural effusion (Figures 1, 2).

**Table 1:** Laboratory findings on admission Laboratory tests showed a mildly elevated C-reactive protein (CRP) level, while white blood cell count and lactate dehydrogenase remained within normal ranges, suggesting the absence of bowel necrosis.

Parameter	Value (SI units)	Reference range
WBC (×10⁹/L)	7.13	3.5–9.0
RBC (×10¹²/L)	3.58	4.3–5.7
Hemoglobin (g/L)	116	135–175
Platelets (×10⁹/L)	169	150–350
Total protein (g/L)	56	66–83
Albumin (g/L)	33	35–50
Total bilirubin (μmol/L)	12	3.4–20.5
Alkaline phosphatase (μkat/L)	0.75	0.50–1.67
Aspartate aminotransferase (μkat/L)	0.317	0.17–0.67
Alanine aminotransferase (μkat/L)	0.15	0.17–0.83
Lactate dehydrogenase (μkat/L)	2.651	2.25–3.75
γ-Glutamyltransferase (μkat/L)	0.3	0.17–0.92
Blood urea nitrogen (mmol/L)	4.64	2.5–7.1
Creatinine (μmol/L)	61	44–106
Estimated GFR (mL/min/1.73 m²)	80	≥60
Sodium (mmol/L)	135	135–145
Potassium (mmol/L)	4.1	3.5–5.0
Chloride (mmol/L)	101	98–107
Amylase (μkat/L)	1.15	0.33–1.67
C-reactive protein (mg/L)	34.1	< 3.0

**Figure 3:**
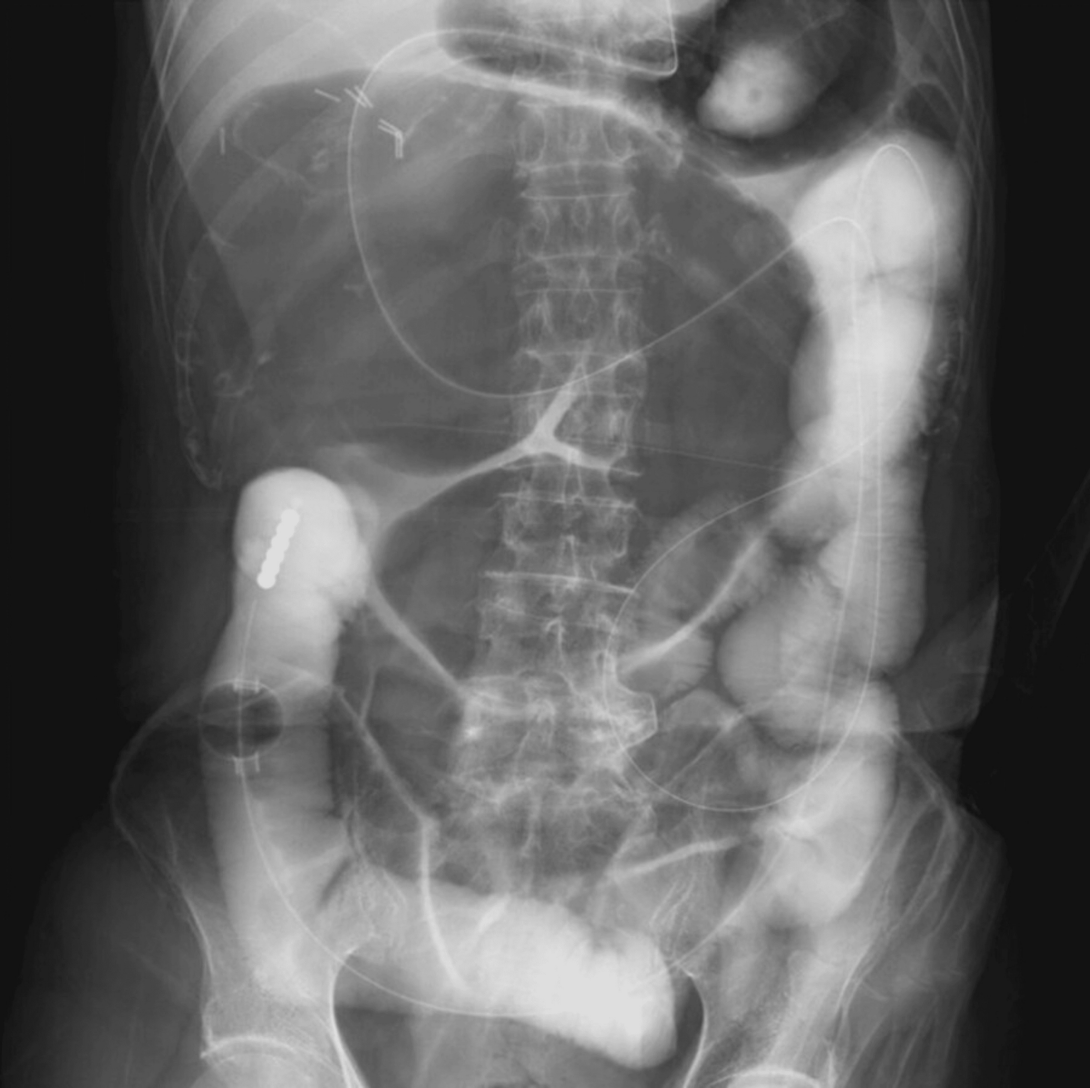
Transnasal ileus tube insertion proximal to the dilated colon Abdominal radiograph showing transnasal ileus tube positioned in the small intestine proximal to the dilated cecum.

**Figure 4:**
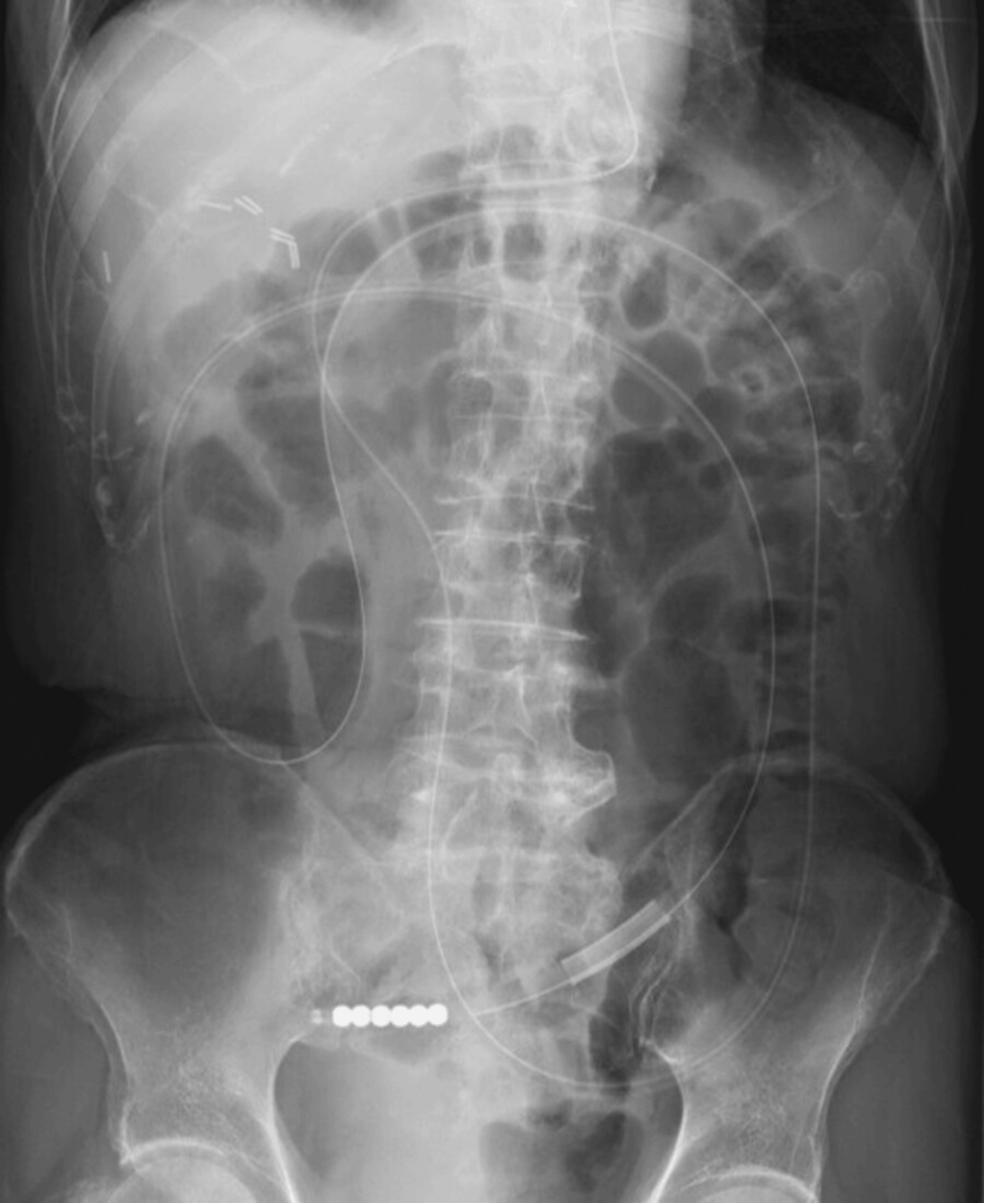
A follow-up abdominal radiograph confirmed resolution of the volvulus Follow-up abdominal radiograph on hospital day 7 showing resolution of the cecal volvulus. The previously dilated cecum has returned to normal caliber, confirming successful decompression after transnasal ileus tube placement.

Given the patient's advanced age and complications, including atelectasis and pleural effusion, emergency surgery was considered high-risk. Since there were no signs of intestinal necrosis, conservative management with transnasal ileus tube decompression was initiated. Under fluoroscopic guidance, the transnasal tube was advanced into the small intestine just proximal to the dilated cecum (Figure 3). Gradual decompression was achieved, with resolution of the volvulus confirmed on day 7 (Figure 4). The tube was removed, and oral intake was resumed thereafter. As the patient tolerated oral intake without issues, he was discharged on hospital day 16. Two weeks post-discharge, elective laparoscopic ileocecal resection was subsequently performed using a five-port approach, with extracorporeal functional end-to-end anastomosis. The patient developed postoperative pneumonia, managed with antibiotics. Apart from this complication, recovery was uneventful, and the patient was discharged on postoperative day 19. At the 11-month follow-up, the patient remained well without recurrence or further complications.

## Discussion

The treatment strategy for cecal volvulus depends primarily on the presence or absence of intestinal necrosis. In cases with any doubts of cecal necrosis, emergency surgical intervention is mandatory, with treatment options including resection with primary anastomosis or resection with stoma formation. Resection with anastomosis has been associated with a reported morbidity of 29%, mortality of 22%, and a recurrence rate of 0% [4]. While data regarding resection with stoma formation are limited, this approach should be considered in high-risk patients for whom anastomosis is not feasible.

Even in the absence of intestinal necrosis, emergency surgery remains the standard treatment. Surgical options include simple detorsion, detorsion with cecopexy, or resection with anastomosis. For prevention of recurrence, detorsion with cecopexy or resection with anastomosis is recommended, with reported recurrence rates of 13% and 0%, respectively [4,5]. Cecopexy is typically reserved for patients considered unsuitable candidates for resection with anastomosis, particularly in consideration of the higher recurrence rate associated with this procedure [5].

Non-operative interventions, such as colonoscopic reduction or decompression via transanal ileus tube insertion, have been reported in selected cases without signs of ischemia. Colonoscopic reduction has a low success rate (14%) and carries a risk of perforation and is not generally recommended [5]. Although definitive evidence supporting the use of transanal ileus tubes in managing cecal volvulus is lacking, transanal ileus tube insertion to the terminal ileum through the dilated cecum has been reported to be effective in the selected case without signs of intestinal ischemia, with resolution of volvulus observed on day 7 following tube placement [3]. It is hypothesized that decompression of the distended colon may facilitate the restoration of peristalsis and lead to spontaneous detorsion. In the present case, a transnasal ileus tube was placed in the small intestine proximal to the distended cecum to achieve proximal decompression. Although there are no previously published reports of transnasal ileus tube insertion for cecal volvulus, if the hypothesized mechanism of decompression-induced detorsion is valid, the transnasal route may represent a similarly effective alternative.

Resolution of the volvulus in the present case was confirmed on an abdominal radiograph seven days after tube insertion. To date, only a single case of non-operative management of cecal volvulus using a transanal ileus tube has been reported [3], and quantitative data regarding success or complication rates are unavailable. The present case demonstrates that transnasal ileus tube insertion can achieve effective decompression while avoiding passage through the fragile, volvulated colonic segment, potentially reducing the risk of colonic perforation. However, transnasal insertion carries a potential risk of vomiting and aspiration during placement, whereas the transanal route is generally considered to carry a lower risk of these complications. The choice between transnasal and transanal approaches should be guided by institutional experience and patient-specific factors, such as age, comorbidities, anatomical considerations, and overall clinical status.

The principal benefit of non-operative decompression in cases of cecal volvulus without signs of intestinal ischemia is the potential to avoid emergency surgery. Emergency surgical procedures are known to carry significantly higher risks of postoperative complications and mortality compared to elective operations. In the present case, the patient’s advanced age and complications such as atelectasis placed him at particularly high risk for emergency surgery. According to the NSQIP surgical risk calculator (American College of Surgeons, Chicago, IL, USA), the estimated complication and mortality rates for emergency partial colectomy with anastomosis were 38.6% and 23.8%, respectively, whereas elective colectomy would reduce these rates to 24.8% and 8.3%, respectively. In addition, the presence of concurrent atelectasis was associated with a 2.3-fold increase in postoperative pneumonia risk [6]. Thus, in high-risk patients such as the present case, avoiding emergency surgery is particularly desirable.

Although there are several reports suggesting that cecal volvulus without intestinal necrosis may be non-operatively resolved through colonoscopic reduction or ileus tube insertion, it must be acknowledged that this approach carries the inherent risk of progression to intestinal necrosis. It has been reported that in cases where intestinal necrosis develops, emergency surgery is associated with a mortality rate more than twice as high as that of emergency surgery performed in the absence of necrosis [7]. Therefore, when considering ileus tube insertion as a conservative treatment strategy, the associated risks must be thoroughly explained to the patient, and the decision should be made with utmost caution. Moreover, surgical intervention should be proactively considered in clinical scenarios indicative of impending ischemia, including progressive cecal dilatation (clinically significant risk of perforation once >9 cm, markedly increased above 12 cm) [8,9], elevation of inflammatory markers or lactate levels, worsening abdominal pain, or the presence of peritoneal signs. Careful integration of radiological findings, laboratory data, and clinical presentation is essential in determining the appropriateness of conservative versus surgical management.

This case highlights that transnasal ileus tube decompression may represent a viable alternative to emergency surgery in carefully selected high-risk patients. While the current evidence is limited, further studies are warranted to evaluate the safety, efficacy, and optimal selection criteria for broader clinical application in cases of cecal volvulus.

## Conclusions

In conclusion, successful decompression and resolution of cecal volvulus without intestinal necrosis were achieved in a high-risk patient through transnasal ileus tube placement, subsequently allowing for elective surgery. These observations suggest that transnasal decompression may serve as a bridge strategy in carefully selected patients. Nevertheless, surgical management remains the established standard of care, and non-operative approaches should be reserved for select circumstances. Further accumulation of clinical experience and systematic investigations will be essential to clarify the safety, efficacy, and appropriate indications for this approach.
